# Simplified quantum optical Stokes observables and Bell’s theorem

**DOI:** 10.1038/s41598-022-14232-8

**Published:** 2022-06-16

**Authors:** Konrad Schlichtholz, Bianka Woloncewicz, Marek Żukowski

**Affiliations:** University of Gdansk, International Centre for Theory of Quantum Technologies (ICTQT), 80-308 Gdańsk, Poland

**Keywords:** Quantum optics, Quantum information, Theoretical physics

## Abstract

We discuss a simplified form of Stokes operators for quantum optical fields that involve the known concept of binning. Behind polarization analyzer photon numbers (more generally intensities) are measured. We have two outputs, say, for horizontal and vertical polarization. If the value obtained in horizontal output is greater than in vertical one we put 1. Otherwise, we put − 1. For equal photon numbers, we put 0. Such observables do not have all properties of the Stokes operators, but can be employed in Bell type measurements, involving polarization analyzers. They are especially handy for states of undefined number of photons, e.g. squeezed vacuum and their realisation is intuitive. We show that our observables can lead to quite robust violations of associated Bell inequalities. We formulate a strongly supported numerically conjecture that one can observe with this approach violations of local realism for the four mode squeezed vacuum for all pumping powers (i.e. gain values).

## Introduction

The discussion about what is the essence of quantumness started with the first attempts of formulating quantum mechanics. With the emblematic paper of Einstein et al.^1^ the problem of completeness of quantum mechanics became a point of discussion among the scientific community. This started with the response by Bohr^2^. Many years later, after the paper of Bell^3^ the challenge of revealing non-classicality, in terms of violation of local realism, has entered the core of contemporary research. All that in the meantime gained in importance with the emergence of quantum information and communication.

The ultimate test of non-classicality is the violation of Bell inequalities. This is now also the essence of testing of device-independent quantum communication protocols. Formulations of Bell’s theorem for situations of fixed numbers of particles have already a vast literature, and well established methods, see e.g. reviews^4–7^. However, if one moves to situations with undefined numbers of particles, still the situation is quite open. This is of course e.g. the case of general quantum optical fields. A lot of approaches are tested.

Polarization entanglement experiments are classic examples of experimental tests of Bell’s inequalities. The two photon experiments are a realization of two qubit-entanglement^8,9^. A deceptively obvious step in the direction towards optical fields of undefined photon numbers is to use quantum Stokes observables. The usual definition of these runs as follows. If one assumes that the intensity of light is proportional to the photon number, then (standard) quantum Stokes observables are given by $\hat{\Theta }_i = \hat{a^{\dagger }}_i\hat{a}_{i} - \hat{a^{\dagger }}_{i\perp }\hat{a}_{i_{\perp }},$ where $\hat{a}$ is an annihilation operator. Indices $i = 1,2,3$ mark three mutually unbiased (fully complementary) polarization analyzers settings. The indexes, *i* and $i_{\perp }$ stand for two orthogonal polarizations. E.g., one might choose the *i*’s to represent horizontal-vertical, $\{H,V\}$, diagonal-antidiagonal, $\{45^{\circ }, -45^{\circ }\}$, or right-left handed circular, $\{R,L\}$, polarization analyzer settings. The zeroth Stokes operator is given by the total photon number operator $\hat{\Theta }_0 = \hat{N}= \hat{a^{\dagger }}_i\hat{a}_{i} + \hat{a^{\dagger }}_{i\perp }\hat{a}_{i_{\perp }}$^10^.

If we are interested in the degree of polarisation of light we use $\big (\frac{\sum _i\langle {\Theta _i}\rangle ^2}{\langle {\Theta _0}\rangle ^2}\big )^{1/2}$. Obviously, this parameter is not a formal quantum observable (a self-adjoint linear operator). Neither is $\frac{\langle {\Theta _i}\rangle }{\langle {\Theta _0}\rangle }$. This is one of the reasons why attempts to build Bell inequalities using such parameters and their correlators for observation stations *A* and *B* in the form of $\frac{\langle {\Theta ^A_i\Theta ^B_j}\rangle }{\langle {\Theta ^A_0\Theta ^B_0}\rangle }$ fail and lead to misleading conclusions^11^. This is because such attempts involve additional assumptions, beyond the usual ones for Bell inequalities, which limit the range of local hidden variable theories for with such Bell inequalities must hold.

Bell inequalities for Stokes parameters can be formulated if one introduces normalized Stokes observables^12–14^:

1 $$\begin{aligned} \hat{S}_{j} = \hat{\Pi }\frac{\hat{n}_{j} - \hat{n}_{{j}_\perp }}{\hat{n}_{j} + \hat{n}_{{j}_\perp }} \hat{\Pi }, \end{aligned}$$

where $\hat{\Pi }={\mathbb {I}}-|{\Omega }\rangle \langle {\Omega }|$, and $|{\Omega }\rangle$ is the vacuum state (of the optical beam in question).

It has been shown that such operators allow for the construction of stronger entanglement criteria, and they are a handy tool for formulation of Bell inequalities. One of their properties, crucial in this case, is the fact that these operators have a spectrum bounded by $-1$ and 1. That is, they have the basic property of observables which allows one to derive the CHSH-Bell inequalities. Thus, a derivation of a version of CHSH inequality applicable for such Stokes operators is essentially a replacement procedure. With the recent development of measurement techniques allowing photon number resolving detection^15,16^ the discussion about normalized Stokes parameters stops to be only theoretical and its use in experiments is becoming feasible.

Note that what makes Pauli operators so straightforwardly applicable to Bell inequalities is their dichotomic nature. One of the attempts to construct field operators of a similar property was the formulation of pseudo-spin operators. For example, the *z* component of pseudo-spin is $(-1)^{\hat{n}}$, where $\hat{n}$ is the total photon number operator in the given optical mode^17,18^. The spectrum of pseudo-spin operators is the same as the spectrum of Pauli matrices, but their use introduces great difficulties from the experimental point of view. Even a loss of one photon (due to e.g. detector inefficiency) or a single dark count reverses the result of a measurement.

Here we analyze a simpler approach, which leads to proper Bell inequalities for polarization measurements of quantum optical fields. Our aim is to construct a family of operators that would have the usual spectrum for Bell experiments and would be robust with respect to experimental noise. We present polarization quantum field observables that have spectrum limited to $\pm 1$ and 0. Our initial ideas on such binning can be found in^19^. The approach to binning presented here is concurrent with the method used in^20^ in the context of correlation in Bose-Einstein condensates. With the observables, we construct Bell inequalities. We test their resilience under losses and noise for $2 \times 2$ mode bright squeezed vacuum and bright GHZ radiation. The observables are realizable in the laboratory with standard measurement devices. They are described in the next section.

## New operators: sign Stokes operators

It was shown that Bell inequalities constructed with normalized Stokes operators can be violated by macroscopic states of light such as $2 \times 2$ (bright) squeezed vacuum (BSV)^14^ and its GHZ-like generalization (BGHZ)^21^. However, for a higher mean number of photons, the violation of Bell inequalities by these states is quickly damped. This results in lowering of the threshold values for pumping strength after which violation cannot be observed.

We address those problems by another normalization scheme, based on the so-called binning, which we call Sign approach normalization. To obtain new operators, we use the sign function and apply it to Stokes operators:

2 $$\begin{aligned} \begin{aligned} \hat{G}(s)&= \mathrm {sign}(\hat{n}_s-\hat{n}_{s_\bot })=\mathrm {sign}(\hat{\Theta }_s)=\mathrm {sign}(\hat{U}_s(\hat{n}_H-\hat{n}_{V})U_s^{\dagger }), \end{aligned} \end{aligned}$$

where *s* denotes the chosen setting related with the corresponding polarization basis with the eingenstates given by *s* and $s_{\bot }$. Subscripts *H* and *V* refer to horizontal and vertical polarizations, and the operator $\hat{U}_s$ is a unitary transformation that transforms the polarization modes *H*, *V* into another orthogonal pair of, in general, elliptic polarization modes $\{s, s_\perp \}$. From (2) we see that the eigenstates of *G*(*s*) are $|{j_s k_{s_{\perp }}}\rangle =\frac{1}{\sqrt{j!k!}}\hat{a}_s^{\dagger j}\hat{a}_{s_\perp }^{\dagger k}|{\Omega }\rangle ,$ where $\hat{a}_s^{\dagger }$ and $\hat{a}_{s_\perp }^{\dagger }$ are creation operators related to the respective polarization modes of the given beam. The spectral form of (2) is given by:

3 $$\begin{aligned} \begin{aligned} \hat{G}(s)=\sum _{k>j}\Big (|{k_s, j_{s_\perp }}\rangle \langle {k_s, j_{s_\perp }}|-|{j_s, k_{s_\perp }}\rangle \langle {j_s, k_{s_\perp }}|\Big ). \end{aligned} \end{aligned}$$

Formula (3) clearly shows that the new operators are well-defined Hermitian operators and that each $\hat{G}(i)$ has three eigenvalues $\pm 1$ and 0. Although formula (2) implies photon number operators, the basic idea of sign as well as standard and normalized Stokes operators is based on differences and sums of intensities. These in turn do not need to be modeled with photon counts. Note that formula (3) does not imply any particular model of intensity as long as the intensity increases with number of counts (even nonlinearly).

The action of the $\mathrm {sign}$ function on Stokes operators can be regarded as some form of the binning strategy used in the context of polarization measurements. Binning strategies are e.g. used in homodyne schemes for observing non-classicality^22–25^.

We shall call the new operators sign Stokes operators. Following the usual approach, we shall define a triad of sign Stokes operators, related to the three maximally complementary settings of a polarization analyzer. For the usual triad of such settings, we denote by $\hat{G}_1$ the sign operator the eigenstates of which refer to $\{s = D, s_{\perp } =A\}$ polarization basis, and by $\hat{G}_2$ and $\hat{G}_3$ for respectively $\{R,L\}$ and $\{H,V\}$ bases. However, this notation is also extended to other triads of maximally complementary settings.

The sign operators share some properties of Stokes and normalized Stokes operators. Importantly, once one has a photon-number-resolving detection setup, the data collected in each run allows one to compute the obtained values of each of Stokes operators for the given basis *i*: standard, normalized, and sign ones, as they depend solely on the measured photon numbers $n_i$ and $n_{i{\perp }}$. As we see, the new approach is in fact just a new form of data analysis that turns out to be simple and efficient. Further, in order to measure different sign operators $\hat{G}_{s'}$, that is, to move from *s* to $s'$, it is enough to change the polarization analysis basis. Being useful from experimental point of view, unfortunately sign Stokes operators do not share all properties of quantum Stokes operators, what puts some limitations on their use in entanglement detection.

### Stokes-like vector cannot be formed out of sign Stokes operators

Standard Stokes operators form a Stokes vector. We will discuss this property for pure states. However, it works also for mixed ones. We have $$\langle \hat{\overrightarrow{\Theta }}\rangle _\psi =(\langle \hat{\Theta }_1\rangle _\psi ,\langle \hat{\Theta }_2\rangle _\psi ,\langle \hat{\Theta }_3\rangle _\psi )$$ where $$|{\psi }\rangle $$ is an arbitrary state of the optical field. The Euclidean norm of this vector fulfills : $$||\langle \overrightarrow{\Theta }\rangle _\psi ||\le \langle {\hat{\Theta }_0}\rangle _{\psi }$$. We can construct an analogue vector for normalized Stokes operators and $$||\langle \hat{\mathbf {S}}\rangle _\psi ||\le \langle {\hat{S}_0}\rangle _{\psi }\le 1$$^13^. These norms remain invariant under any unitary transformation between two triads of mutually maximally complementary polarization analysers. This transformation can be put as $$a_{3'}= U_{11}a_{3}+U_{12}a_{3_\perp }$$ and $$a_{{3'}_{\perp }}= U_{21}a_{3}+U_{22}a_{3_\perp }$$, where $$ U_{ij}$$ are elements of a certain two-dimensional unitary matrix. Properly defined Stokes vector has its Euclidean norm invariant with respect to such mode transformations. As a transformation of this kind can also be expressed as a transformation of the state, one can introduce $$|{\psi '}\rangle $$, which is in the following relation with $$|{\psi }\rangle $$. If $$|{\psi }\rangle = f(a^\dagger _3,a^\dagger _{3_\perp })|{\Omega }\rangle $$, then $$f(a^\dagger _{3'},a^\dagger _{{3'}_\perp })|{\Omega }\rangle =|{\psi '}\rangle $$, where *f*(*x*, *y*) is a polynomial of both variables. We put this relation as $$|{\psi '}\rangle = \hat{U}^\dagger _{mode}|{\psi }\rangle , $$ as it is obviously a specific unitary transformation of the state.

For such mode transformations we have $||\langle \hat{\overrightarrow{\Theta }}\rangle _\psi ||=||\langle \hat{\overrightarrow{\Theta }}\rangle _{\psi '}||$ and $||\langle \hat{\overrightarrow{S}}\rangle _\psi ||=||\langle \hat{\overrightarrow{S}}\rangle _{\psi '}||$. The norm of Stokes vectors, standard and normalized, is constant under any unitary transformation of the triads polarization analysis bases. These features of Stokes observables play a key role in the construction of entanglement indicators involving Stokes operators.

Such properties are not shared by sign operators. Let us construct $\langle \hat{ \overrightarrow{G}}\rangle _\psi = (\langle \hat{G}_1\rangle _\psi ,\langle \hat{G}_2\rangle _\psi ,\langle \hat{G}_3\rangle _\psi )$. It can be shown that $||\langle \overrightarrow{G}\rangle _\psi || \ne ||\langle \overrightarrow{G}\rangle _{\psi '}||$. It is enough to find one counterexample. Consider the state $|{\psi }\rangle = |{3_H,0_V}\rangle$ i.e. the Fock state with 3 photons polarized horizontally. It can be easily checked that for this state $||\langle \overrightarrow{G}\rangle _{\psi }||=1$. Now let us apply a unitary transformation on optical modes of $|{\psi }\rangle$ such that the creation operators transform as follows: $\hat{a}^{\dagger }_H \rightarrow \hat{a}^{\dagger }(\alpha ) = \cos \alpha \hat{a}^{\dagger }_H + \sin \alpha \hat{a}^{\dagger }_V$ and $\hat{a}^{\dagger }_V \rightarrow \hat{a}^{\dagger }_{\perp }(\alpha ) = -\sin \alpha \hat{a}^{\dagger }_H + \cos \alpha \hat{a}^{\dagger }_V$. Let $\alpha = \pi /8$. One gets: $||\langle \overrightarrow{G}\rangle _{\psi '}|| \approx 1,5$. Thus, the norm is not an invariant of the unitary transformations, and additionally it is not bounded by 1. This fact prohibits one to use methods of construction of entanglement indicators presented in^13^, which work via a simple replacement of Pauli operators in entanglement conditions for qubits, by Stokes operators, standard or normalized. Still, as we shall see, there is no obstacle to using this method in the case of construction of Bell inequalities.

Rotational *covariance* of polarization variables is not a necessary feature required to derive Bell inequalities (however, see^26^ for the consequences of demanding exactly that). This allows one to construct CHSH and CH inequalities for fields with sign Stokes observables.

### CHSH inequality

To derive Bell inequalities satisfied by any local realistic description, we start by defining local hidden values that predetermine the output of the measurement of sign Stokes operators (2). We denote the local hidden variables by $$\lambda $$. The functions $$I^X(s,\lambda )$$ and $$I^X(s_{\perp },\lambda )$$ give the predetermined outcomes of the intensity measurements of polarizations *s*, $$s_\perp $$ in the local beam for the observer *X*. We define the local hidden values for sign operators as $$G^X(s,\lambda )=\mathrm {sign}(I^X(s,\lambda )-I^X(s_\perp ,\lambda ))$$. These local hidden values are $$\pm 1$$ and 0, thus one can use standard methods to derive CHSH inequality. The alternative settings will be denoted here by $$ s, s'$$ for the first observer and $$r, r'$$ for the second observer. The resulting CHSH inequality reads:

4 $$\begin{aligned} \begin{aligned} |\langle G^{1}(s,\lambda ) G^{2}(r,\lambda )+ G^{1}(s,\lambda ) G^{2}(r',\lambda )+ G^{1}(s',\lambda ) G^{2}(r,\lambda )- G^{1}(s',\lambda ) G^{2}(r',\lambda )\rangle _{LHV}|\le 2. \end{aligned} \end{aligned}$$

For further reference, we put it as $|CHSH_{G}|\le 2$.

However, this inequality cannot be violated by states with a significant vacuum component, e.g. the (polarization) four-mode squeezed vacuum state, which will be our working example, see next sections. This situation is analogous to the case of normalized Stokes operators, see^14^. Following ideas of^14^ we modify sign Stokes operators as follows:

5 $$\begin{aligned} \hat{G}^X(s)\rightarrow \hat{G}^{X-}(s)=\hat{G}^X(s)-\hat{\Pi }_{\Omega ^X}, \end{aligned}$$

where $\hat{\Pi }_{\Omega ^X}$ is the projector on the subspace of the Fock space of states with no photons in the local beam. Such a projection allows for reduction of the impact of vacuum term, which often appears with the highest probability. Also local hidden values need to be modified:

- $$G^{X-}(s,\lambda )=\mathrm {sign}(I^X(s,\lambda )-I^X(s_\perp ,\lambda ))$$ if $$I^X(s,\lambda )+I^X(s_\perp ,\lambda )\ne 0$$
- $$G^{X-}(s,\lambda )=-1$$ if $$I^X(s,\lambda )+I^X(s_\perp ,\lambda )=0$$

As this modification does not change local hidden values $G^{X-}(s,\lambda ) \in \{0, \pm 1\}$ we use the following CHSH inequality:

6 $$\begin{aligned} \begin{aligned} |CHSH_{G-}|= |&\langle G^{1-}(s,\lambda ) G^{2-}(r,\lambda )+ G^{1-}(s,\lambda ) G^{2-}(r',\lambda )\\&+G^{1-}(s',\lambda ) G^{2-}(r,\lambda )- G^{1-}(s',\lambda ) G^{2-}(r',\lambda )\rangle _{LHV}| \le 2. \end{aligned} \end{aligned}$$

**Figure 1:**
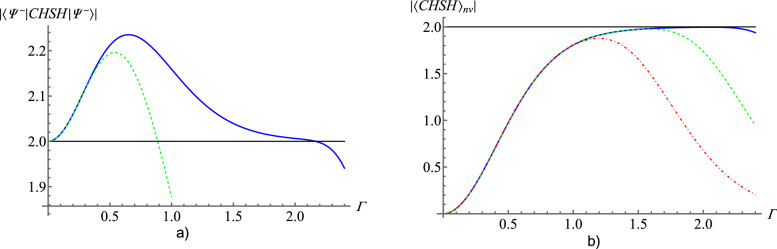
(**a**) The blue curve: the value of the $CHSH_{G-}$ expression based on sign operators, see (6), and the green dashed curve: $CHSH_{S-}$ based on normalized Stokes operators^14^ in a function of amplification gain $\Gamma$ of the *BSV* state. The numerical results were obtained with a cut-off of the expansion of the BSV state at the term $|{\psi ^{n=150}}\rangle$. The maximal values of amplification gain ($\Gamma _{tr}$), such that for all $\Gamma < \Gamma _{tr}$ CHSH inequalities are violated, are $\Gamma _{tr} \approx 0.88$ for normalized Stokes operators^14^ and $\Gamma _{tr} \approx 2.16$ for sign Stokes operators. Thus, with sign Stokes operators, the range of violation with respect to amplification gain is much larger than in the case of normalized Stokes operators. (**b**) The graphs show the non-vacuum term of $CHSH_{G-}$ as a function of amplification gain $\Gamma$ for the BSV state, which was computed for cutoffs of 15, 47, 150 photons. This is done to illustrate that the descent of the curves for high $\Gamma$’s is an artefact of the applied cutoff. The blue curve represents calculations with the cutoff at 150 photons,for the green dashed curve it is at 47 photons and for the red dot-dashed curve at 15 photons. The cutoff seems to be responsible for the decrease of the value in (**a**) for high $\Gamma$’s.

#### Violation of Bell inequality for four mode squeezed vacuum—asymptotic behaviour

We are going to analyze how the use of sign Stokes operators in CHSH inequality helps to reveal the non-classicality of quantum states. Our working example is $2\times 2$ mode squeezed vacuum state (BSV) which is the generalization of EPR singlet. It reads:

7 $$\begin{aligned} |{\psi _-}\rangle = \frac{1}{\cosh ^2(\Gamma )}\sum _{n=0}^\infty \frac{\tanh ^n(\Gamma )}{n!} (a_H^\dagger b_V^{\dagger }-a_V^\dagger b_H^{\dagger })^n |{\Omega }\rangle = \frac{1}{\cosh ^2(\Gamma )}\sum _{n=0}^\infty \sqrt{n+1}\tanh ^n(\Gamma )|{\psi ^n}\rangle , \end{aligned}$$

where $\Gamma$ is the amplification gain and

8 $$\begin{aligned} \begin{aligned} |{\psi ^n}\rangle =&\frac{1}{\sqrt{n+1}}\sum _{m=0}^n(-1)^m|{{(n-m)}_{H_1},{m}_{V_1},{m}_{H_2},{(n-m)}_{V_2}}\rangle . \end{aligned} \end{aligned}$$

Subscripts $H_{1_{(2)}}$ and $V_{1_{(2)}}$ specify the polarization of each mode and to which of the two optical beams it corresponds. We use the convention that $a^\dagger _s$ denotes the creation operator for the photon heading observer *A*, and $b^\dagger _s$ is the creation operator related to the observer *B*. The amplification gain determines the intensity of the pumping field and thus $\Gamma$ sets the expectation value of the intensity of the BSV state.

Assume that both observers choose to measure only linear polarizations. thus, the angles by which the measurement polarization basis is rotated with respect to $\{H,V\}$ basis define the settings. With the notation used in “Stokes-like vector cannot be formed out of sign Stokes operators” section for unitary transformation between linear polarization modes we chose for the first observer $\alpha _s=0$, $\alpha _{s'}=\pi /4$, and for the second one $\alpha _r=\pi /8$ and $\alpha _{r'}=-\pi /8$. It was shown that these settings are optimal in case of violation of CHSH inequality with normalized Stokes operators for BSV^14^.

Figure 1 shows quantum predictions for $CHSH_{G-}$ (6) and the values of CHSH expression for normalized Stokes parameters for BSV taken from^14^ as a function of the amplification gain $\Gamma$. Sign Stokes operators give $|\langle {\psi _-}|CHSH_{G-}|{\psi _-}\rangle |>2$ for a wider range of an amplification gain that is up to $\Gamma _{tr}\approx 2.16$. For normalized Stokes operators, this maximal value of amplification gain is significantly lower, i.e. $\Gamma _{tr} \approx 0.8866$. Thus, with sign Stokes operators it is possible to reveal the non-classicality of BSV for a much higher value of amplification gain.

In Fig. 1 we can see that for $\Gamma \approx 2.1$ for sign Stokes operators $|CHSH_{G-}|$ drops down suspiciously suddenly. We presume that such behaviour might be a consequence of a cut-off. The expansion of $|{\psi _-}\rangle$ was cut off in the numerical calculations at $|{\psi ^{n=150}}\rangle$. This still requires further investigation.

Because of the rotational invariance of $|{\psi _-}\rangle$, it is a “super-singlet”, the expectation values of the correlators entering the Bell inequalities depend, if we measure linear polarizations on both sides, only on *relative angle* of the orientation of the polarization analyzers at the two spatially separated observation stations.

Note that standard, normalized, and sign Stokes operators are composed of functions of photon number operators, which do not change the number of photons. Thus, the expression $\langle {\psi _-}|CHSH_{G-}|{\psi _-}\rangle$ consists of two terms: *vacuum term*, that is CHSH inequality averaged over the vacuum component of BSV and *non-vacuum term*. The vacuum and non-vacuum terms in (6) for our settings are both negative. That is why we can consider the CHSH inequality in question as the sum of absolute values of these both terms. The vacuum term can be easily calculated:

9 $$\begin{aligned} |\langle {\Omega }|CHSH_{G-}|{\Omega }\rangle | = \frac{2}{\cosh ^4\Gamma }. \end{aligned}$$

The non-vacuum term $\langle CHSH_{G-}\rangle _{nv}=\langle {\psi _-}|CHSH_{G-}|{\psi _-}\rangle -\langle {\Omega }|CHSH_{G-}|{\Omega }\rangle$ results from the expectation values of $|{\psi ^n}\rangle$. Note that as $\Gamma$ increases, the role of non-vacuum terms in $\langle {\psi _-}|CHSH_{G-}|{\psi _-}\rangle$ increases too. For small $\Gamma$ the contribution of vacuum term is dominant.

In Fig. 1 the value of the non-vacuum $|\langle CHSH_{G-}\rangle _{nv}|$ is presented. The calculation is performed for BSV state truncated to $n=150$, blue curve, $n=47$, green curve, and $n=15$, red curve. These numbers increase approximately as a geometrical sequence by $\sqrt{10}$ what allows as to analyze the behaviour of $\langle CHSH_{G-}\rangle _{nv}$ within the whole order of magnitude. All curves asymptotically go to 2 (classical bound) up to some point for which they both start to decrease. Note that the curves for $n=15$ and $n=47$ start to decrease for smaller $\Gamma$ than the curve for $n=150$. It is highly probable that the decrease is conditioned by not including components with a high enough number of photons and the non-vacuum term $|\langle CHSH_{G-}\rangle _{nv}|$ goes asymptotically to 2 from the left. The vacuum term goes asymptotically to 0 from the right, see (9). Thus, our hypothesis is that CHSH inequality with sign Stokes operators is violated for BSV for any $\Gamma$. In Supplementary Discussion A we present a reasoning, based on a numerical calculation, supporting this conjecture.

### CHSH inequality with losses

One of the crucial aspects of experimental realization of Bell experiments is detectors with high efficiency $\eta$. Here, we will analyze the critical value of efficiency $\eta _{c}$ such that for $\eta <\eta _{c}$ one cannot observe a violation of (6). We model inefficient detectors in the standard way: a perfect detector ($\eta =1$) with a beamspliter with transmissivity $\sqrt{\eta }$ in front of it. We denote by *k* the number of photons that reach the beamsplitter. Of these, only $\kappa \le k$ counts are registered due to losses on the beamspliter. The probability of registration of $\kappa$ photons is given by the binomial distribution:

10 $$\begin{aligned} p(\kappa |k)= {k\atopwithdelims ()\kappa }\eta ^{\kappa }(1-\eta )^{k-\kappa }. \end{aligned}$$

**Figure 2:**
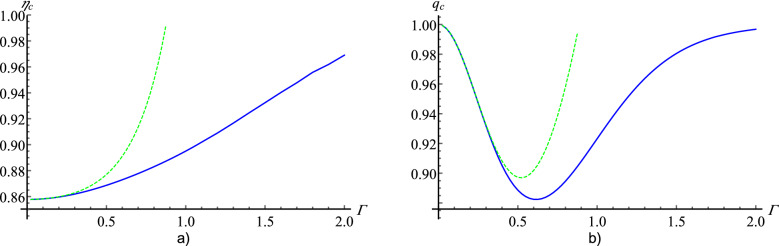
(**a**) Critical efficiency $\eta _c$ versus $\Gamma$ for the CHSH inequalities for the BSV state. A blue curve represents $\eta _c$ for sign approach and a green dashed curve for normalized Stokes operators. (**b**) Critical value of *q* versus $\Gamma$ for the BSV state. A blue curve represents $q_c$ for sign approach and a green dashed curve for normalized stokes operators. Assuming that asymptotic behaviour of violation of CHSH inequality for sing parameters discussed in “Violation of Bell inequality for four mode squeezed vacuum—asymptotic behaviour” section is correct the $q_c$ for the sign Stokes operators goes to 1 in the limit $\Gamma \rightarrow \infty$.

In Fig. 2 we can see the minimal value of efficiency $\eta _c$ for which the violation of CHSH inequality can be observed for normalized and sign Stokes operators in function of $\Gamma$. Note that for small $\Gamma$ (up to $\Gamma \approx 0.3$) the curves for sign and normalized Stokes operators behave almost identically. However, as $\Gamma$ increases, the value of $\eta _c$ for sign Stokes operators grows slower than that for normalized ones. Such a change in rate of growth for a higher $\Gamma$ should be expected because, for a high number of photons, loss of one photon matters less in the case of sign Stokes operators.

### CHSH inequality with noise

In a realistic scenario of a Bell experiment apart from photon losses one shall consider also noise. Our noise is modeled in the similar way as “white noise” for qubits. Let us introduce four squeezed vacuum states which are related with the Bell state basis for two qubits^27^:

11 $$\begin{aligned} |{\Phi ^{\pm }}\rangle = \frac{1}{\cosh ^2(\Gamma )}\sum _{n=0}^\infty \frac{\tanh ^n(\Gamma )}{n!} (a_H^\dagger b_H^{\dagger }\pm a_V^\dagger b_V^{\dagger })^n |{\Omega }\rangle , \end{aligned}$$

and

12 $$\begin{aligned} |{\Psi ^{\pm }}\rangle = \frac{1}{\cosh ^2(\Gamma )}\sum _{n=0}^\infty \frac{\tanh ^n(\Gamma )}{n!} (a_H^\dagger b_V^{\dagger }\pm a_V^\dagger b_H^{\dagger })^n |{\Omega }\rangle . \end{aligned}$$

Our noise model can be defined as follows:

13 $$\begin{aligned} \rho _{noise} = \frac{1}{4}(|{\phi ^+}\rangle \langle {\phi ^+}|+|{\phi ^-}\rangle \langle {\phi ^-}|+|{\psi ^+}\rangle \langle {\psi ^+}|+|{\psi ^-}\rangle \langle {\psi ^-}|). \end{aligned}$$

Note that $\rho _{noise}$ is uncorrelated. Let *q* be the visibility. The noisy state reads:

14 $$\begin{aligned} \begin{aligned} \rho '=q|{BSV}\rangle \langle {BSV}|+ (1-q)\rho _{noise}, \end{aligned} \end{aligned}$$

The value $1-q$ determines the probability of registering noise. Figure 2 shows the minimal value of visibility $q_c$ that ensures the violation of CHSH inequality for normalized and Stokes operators. We see that sign Stokes operators have a similar advantage over normalized Stokes operators as in the case of losses, i.e. for small $\Gamma$ normalized and sign Stokes operators are similarly resistant to noise. As $\Gamma$ increases sign Stokes operators result to be significantly more efficient, Moreover from the results shown on Fig. 2 and the reasoning presented in “Violation of Bell inequality for four mode squeezed vacuum—asymptotic behaviour” section we can conclude that $q_c \rightarrow 1$ when $\Gamma \rightarrow \infty$.

### CH inequality

Going along with the idea of sign operators and rate approach to CH inequality^14^ we can construct a new CH inequality for quantum optical fields. Let us move directly to the quantum scenario and start with the CH operator ($CH_R$) for intensity rates. In^14^ the rates are defined by $\hat{R}_{+}(s)=\hat{\Pi }\hat{n}_s/(\hat{n}_s+n_{s_{\perp }})\hat{\Pi }$. Note that such an operator is simply the first term of normalized Stokes operator (1). Its eigenvalues are rational numbers in (1/2, 1] for photon number states $|{n_s,m_{s_\bot }}\rangle$ where $n>m$ and in [0, 1/2) for states where $n<m$. If $m=n$ the eigenvalue of the rates is 1/2. Combining the idea CH inequality for rates and the concept sign Stokes operators we construct operators for CH inequality based binning. We seek for operators of eigenvalues with the following properties: we have 1 when $m>n$ and 0 if $n\le m$. Such a dichotomic observable is simply a projector onto subspace $n>m$:

15 $$\begin{aligned} \hat{P}(s)=\sum _{n>m}|{n_s,m_{s_\bot }}\rangle \langle {n_s,m_{s_\bot }}|. \end{aligned}$$

The expectation value of $\hat{P}^X(s)$ is equal to the probability that the observer *X* will see $n>m$. We shall denote by $\langle \hat{P}^X(j) \hat{P}^Y(k)\rangle$ the quantum joint probability of obtaining the same result $n>m$ by observers *X* and *Y* for their respective polarization basis *j* and *k*. Had these probabilities in the experiment been classical, and if the assumptions of local realism hold Clauser–Horne inequality tailored for the quantum scenario is given by:

16 $$\begin{aligned} \begin{aligned} -1\le&\langle CH_P\rangle =\Big \langle \hat{P}^1_+(\theta )\hat{P}^2_+(\phi )+\hat{P}^1_+(\theta )\hat{P}^2_+(\phi ')+\hat{P}^1_+(\theta ')\hat{P}^2_+(\phi )-\hat{P}^1_+(\theta ')\hat{P}^2_+(\phi ')-\hat{P}^1_+(\theta )-\hat{P}^1_+(\phi )\Big \rangle \le 0. \end{aligned} \end{aligned}$$

**Figure 3:**
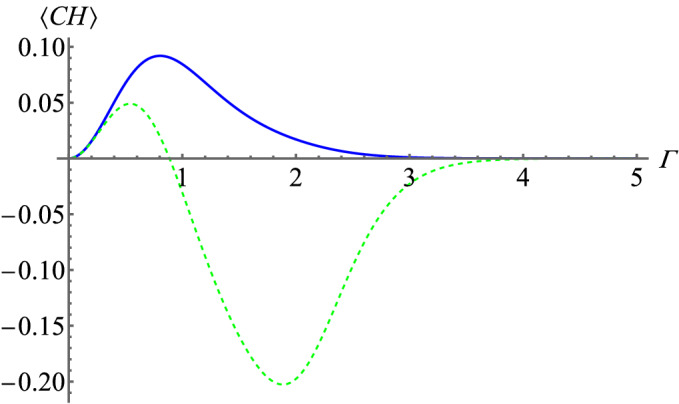
Quantum predictions for expectation value of CH expression for the ‘sign’ approach (blue curve) and rate approach^14^ (green dashed curve) as a function of the amplification gain $\Gamma$ for BSV state. The numerical results were obtained with a cut-off of the expansion of the BSV state at the term $|{\psi ^{n=50}}\rangle$. The upper bound of CH inequality for the ‘sign’ approach is violated in the whole range of $\Gamma$ covered in the figure, while the violation of the inequality in the case of the normalized Stokes operators is quickly damped and after that CH expression goes asymptotically from bellow to the classical bound.

Figure 3 shows the expectation value of the CH expression (16) and its rate counterpart for the same settings as in the case of CHSH inequality. The ‘sign’ approach gives violation of upper bound of CH expression for all $\Gamma$ while the rate approach gives a violation only for $\Gamma <0.8866$ which is the same case as for CHSH. Note that this CH inequality is not equivalent to CHSH inequality (6) (see Supplementary Discussion A)

## Violation of Bell inequalities with sign approach for Bright GHZ state

As another example, let us consider a Bright GHZ state which is a generalization of the two beam squeezed vacuum considered above, to three beam emissions.

Such a process for years was thought to be infeasible, but current experimental progress allows one to think of such a possibility. The usual parametric approximation of the theoretical description of generation process of such states, which describes the pumping field as classical, does not work because of the divergence of perturbation series. Still, with an employment of a version of Padè approximation one can find an approximate parametric description, with convergent perturbation series, see^21^. The approximation gives a state of the following form:

17 $$\begin{aligned} \begin{aligned} |{BGHZ}\rangle =\sum _{k=0}^\infty \sum _{m=0}^k C_{k-m}(\Gamma )C_m(\Gamma )(\hat{a}_i^\dagger \hat{b}_i^\dagger \hat{c}_i^\dagger )^{k-m}(\hat{a}_{i_\perp }^\dagger \hat{b}_{i_\perp }^\dagger \hat{c}_{i_\perp }^\dagger )^m|{\Omega }\rangle . \end{aligned} \end{aligned}$$

The method of obtaining the coefficients $C_m(\Gamma )$ can be found in^21^, and we base our numerical computations on the results established in this reference. The symbols $\hat{a}_p^\dagger$, $\hat{b}_p^\dagger$ and $\hat{c}_p^\dagger$ stand for creation operators in two orthogonal polarization modes $p=i,i_\perp$, of a beam which goes to respectively observers *A*, *B* and *C*. For simplicity, we assumed the polarization modes to be *H*, *V*, that is, $i=3$.

### Mermin-like inequality

Let us consider Mermin-like inequality for quantum optical fields^21^:

18 $$\begin{aligned} |M(3)_{S}|=|\langle S_1^1(\lambda ) S_1^2(\lambda ) S_1^3(\lambda )- S_1^1(\lambda )S_2^2(\lambda ) S_2^3(\lambda )- S_2^1(\lambda ) S_1^2(\lambda ) S_2^3(\lambda )- S_2^1(\lambda ) S_2^2(\lambda ) S_1^3(\lambda )\rangle _{LHV}|\le 2, \end{aligned}$$

where $S_i^X(\lambda )$ are local hidden values corresponding to normalized Stokes operators with polarization bases: $\{45^{\circ }, -45^{\circ }\}$, $\{R,L\}$, for $i=1,2$ respectively. The observers are now marked by $X=1,2,3$. The inequality (18) generalizes Mermin inequality for three qubits^28^ for three photon beams with two polarisation modes each from a parametric source, for details see:^21^. Of course, in general the settings 1, 2 could be different.

The derivation of this inequality requires only that local hidden values are bounded by $\pm 1$. Because local hidden values for sign Stokes operators fulfil this requirement, we can replace $S_i^X(\lambda )$ by $G_i^X(\lambda )$ and obtain a new inequality

19 $$\begin{aligned}&|M(3)_{G}|=|\langle G_1^1(\lambda ) G_1^2(\lambda ) G_1^3(\lambda )- G_1^1(\lambda )G_2^2(\lambda ) G_2^3(\lambda )- G_2^1(\lambda ) G_1^2(\lambda ) G_2^3(\lambda )- G_2^1(\lambda ) G_2^2(\lambda ) G_1^3(\lambda )\rangle _{LHV}|\le 2.&\end{aligned}$$

However, this inequality is not violated by the BGHZ state. We have to again modify sign Stokes operators (as well as normalized Stokes operators):

20 $$\begin{aligned} \hat{G}_i^X\rightarrow \hat{G}_i^{X-}=\hat{G}_i^X-\hat{\Pi }_{\Omega ^X}. \end{aligned}$$

One can easily write modified local hidden values for such operators as in “CHSH inequality” section and obtain inequality:

21 $$\begin{aligned} \begin{aligned}{}|M(3)_{G-}|&= |\langle G_1^{1-}(\lambda )G_1^{2-}(\lambda )G_1^{3-}(\lambda ) - G_1^{1-}(\lambda )G_2^{2-}(\lambda )G_2^{3-}(\lambda )\\ {}&\quad - G_2^{1-}(\lambda )G_1^{2-}(\lambda )G_2^{3-}(\lambda ) - G_2^{1-}(\lambda )G_2^{2-}(\lambda )G_1^{3-}(\lambda )\rangle _{LHV}|\le 2. \end{aligned} \end{aligned}$$

**Figure 4:**
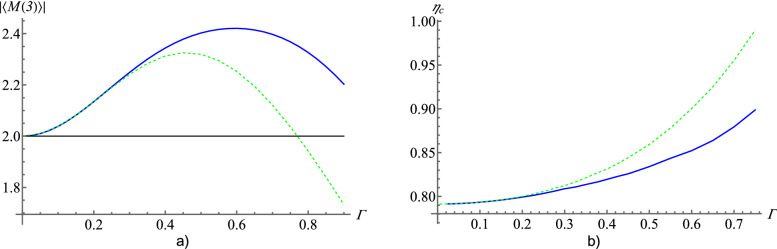
(**a**) Quantum values of $|M(3)_{G-}|$ expression (blue curve) and $|M(3)_{S-}|$ (green dashed curve) as a function of the amplification gain $\Gamma$ for BGHZ state. (**b**) Critical efficiency $\eta _c$ versus $\Gamma$ for Mermin inequalities for the BGHZ state. The blue curve represents $\eta _c$ for the sign approach and a green dashed curve for normalized Stokes operators.

Figure 4 presents quantum values of $|\langle {BGHZ}|\hat{M}(3)_{G-}|{BGHZ}\rangle |$ and of analogous expression, $|\langle {BGHZ}|\hat{M}(3)_{S-}|{BGHZ}\rangle |$, for a Mermin inequality for modified normalized Stokes operators, $\hat{S}_i^{X-}=\hat{S}_i^X-\hat{\Pi }_{\Omega ^X},$ which is of the form (18) with $\hat{S}_i^{X-}$ replacing $\hat{S}_i^{X}$. All that is with respect to the amplification gain $\Gamma$. The range of $\Gamma$ for which the inequality is violated by BGHZ state in the case of sign Stokes operators exceeds the range of applicability of the method used to approximate the probability amplitudes for BGHZ state. We also stress that this result is more robust than in the case of normalized Stokes operators. The graphs in Fig. 4 are discontinued at $\Gamma =0.9$ because for higher values the approximation of ref.^21^ breaks down.

### Mermin-like inequality with losses

We use the model of losses due to inefficient detectors as in “CHSH inequality with losses” section for the inequality (21). In Fig. 4 critical values of efficiency of detectors $\eta _{c}$, for sign and normalized Stokes operators, are compared. We can see that for small $\Gamma$ inequalities exhibit similar resistance to losses. However, with increasing $\Gamma$ difference between the performance of sign and normalized Stokes observables increases in favour of the former ones.

## Conclusions and some open questions

We have proposed, based on a version of the binning approach^19,20^, new Stokes-like polarization observables for quantum optical fields which have a clear operational meaning. In presented examples, the sign Stokes observables allow observation of Bell non-classicality of squeezed-vacuum-type states for pumping powers, for which normalized Stokes observables fail to do so. Sign Stokes operators are easier in experimental realization than normalized ones. Also, they are more resistant to imperfect detection and presence of a noise. One could be tempted to use sign Stokes observables to derive entanglement indicators not based on Bell inequalities. However, such Stokes observables do not possess properties which are commonly used in derivations of bounds for separable states. Simply a triad of them does not form a Stokes vector with proper covariance properties. Thus, this requires a different approach. Similar questions arise when one thinks of a steering condition involving sign Stokes observables.

Another question would be if there is a type of state for which normalized Stokes operators allow for violating of some Bell inequality and for which this is impossible using sign Stokes operators.

The presented results give a possible way to search for violations of local realism in situations with undefined particle numbers, which are so common in especially quantum optics. The associated Bell inequalities are correctly defined. That is, the sole assumption is local realism (and tacitly freedom of the choice of the random settings for all observers involved). No additional “reasonable” assumptions are used. As, according to our numerical estimates, one can conjecture that the associated inequalities are violated for an arbitrary $\Gamma$, they may serve as tool to reveal Bell non-classicality of bright quantum optical states, see^29^. This indicates that such states may find an application in, e.g. quantum communication, provided one finds new suitable Bell inequalities which would lead to more robust violations of local realism.

## Supplementary Information

Supplementary Information.
