# Supplementary material for: Simplified quantum optical Stokes observables and Bell’s theorem
Source: Sci Rep. 2022 Jun 16;12:10101. doi: 10.1038/s41598-022-14232-8 (PMC9203794; doi:10.1038/s41598-022-14232-8)
Supplement: Supplementary file 1 — Supplementary Information. [file 41598_2022_14232_MOESM1_ESM.pdf]

Simplified Quantum Optical Stokes observables and Bell's Theorem - Supplementary Discussion

Konrad Schlichtholz[1,*], Bianka Woloncewicz[1,], and Marek Żukowski[1]**

[1], University of Gdansk, International Centre for Theory of Quantum Technologies (ICTQT), Gdansk, 80-308, Poland
[*]konrad.schlichtholz@phdstud.ug.edu.pl
[**]bianka.woloncewicz@phdstud.ug.edu.pl

1 Supplementary Discussion A: Asymptotic violation of CHSH inequality

Here we present the numerical argumentation for our hypothesis that CHSH inequality (6) with sign Stokes operators is violated for BSV for any Γ.

Let us start with the analysis of quantum predictions for expectation values of (6) for states $|\psi^n\rangle$ i.e. $|\langle CHSH_{G-}\rangle_{\psi^n}|$, for $n \neq 0$, and compare them with analogue expression for normalized Stokes operators.

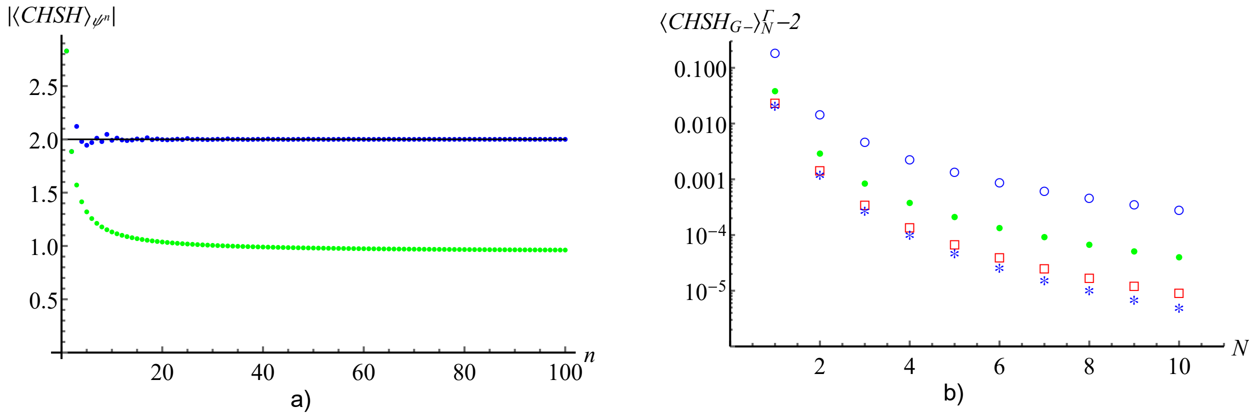

Figure 1. a) $\langle CHSH_{G-}\rangle_{\psi^n}$ for sign Stokes operators (blue points) and analogue for normalized Stokes operators $\langle CHSH_{S-}\rangle_{\psi^n}$ (green points) operators for $|\psi^n_-\rangle$ as a function of n. The points: $n=1$ and $n=2$ of both approaches coincide. For normalized Stokes operators only singlet state contribute to the violation and all points converge to 1 which is the bound for separable states. In the case of sign Stokes operators all points are concentrated around the classical bound for CHSH inequality. b) $\langle CHSH_{G-}\rangle_N^{(\Gamma)}$ versus N. $\Gamma=1$ circles, $\Gamma=2$ dots, $\Gamma=3$ squares, $\Gamma\to\infty$ stars. For any Γ values of $\langle CHSH_{G-}\rangle_N^{(\Gamma)}$ go to 2 with growing N. The case of $\Gamma\to\infty$ bounds $\langle CHSH_{G-}\rangle_N^{(\Gamma)}$ from bellow.

FIG.1 shows results for $n=1,\ldots,100$ for sign Stokes operators and normalized Stokes operators. For normalized Stokes operators only for $n=1$ we get $|\langle CHSH_{S-}\rangle_{\psi^n}| \geq 2$. For sign operators, the values of $|\langle CHSH_{G-}\rangle_{\psi^n}|$ concentrate around 2 with growing n. More detailed analysis (see FIG. 2) reveals two patterns: an oscillating one for odd n's and a pattern converging to 2 from below for even n's. The period of odd n's is equal to $T=8$ in the sense that points $n=2k+1$ and $n=2k+1+T$ where $k\in\mathbb{N}$ corresponds to e.g. two adjacent maximums in the pattern. The even pattern also has an internal structure repeatable with $T=8$.

The periodicity of the pattern values being above and below 2 provides us the natural grouping of $|\langle CHSH_{G-}\rangle_{\psi^n}|$. Let us examine a weighted average $\langle CHSH_{G-}\rangle_N^{(\Gamma)}$ of $|\langle CHSH_{G-}\rangle_{\psi^n}|$ for a given Γ over N-th period for $\Gamma>2$:

$$\langle CHSH_{G-}\rangle_N^{(\Gamma)} = \frac{\sum_{n=1+T(N-1)}^{T+T(N-1)} |\langle CHSH_{G-}\rangle_{\psi^n}|\,|\langle\psi^n_-|\psi_-\rangle|^2}{\sum_{n=1+T(N-1)}^{T+T(N-1)} |\langle\psi^n_-|\psi_-\rangle|^2}, \tag{A1}$$

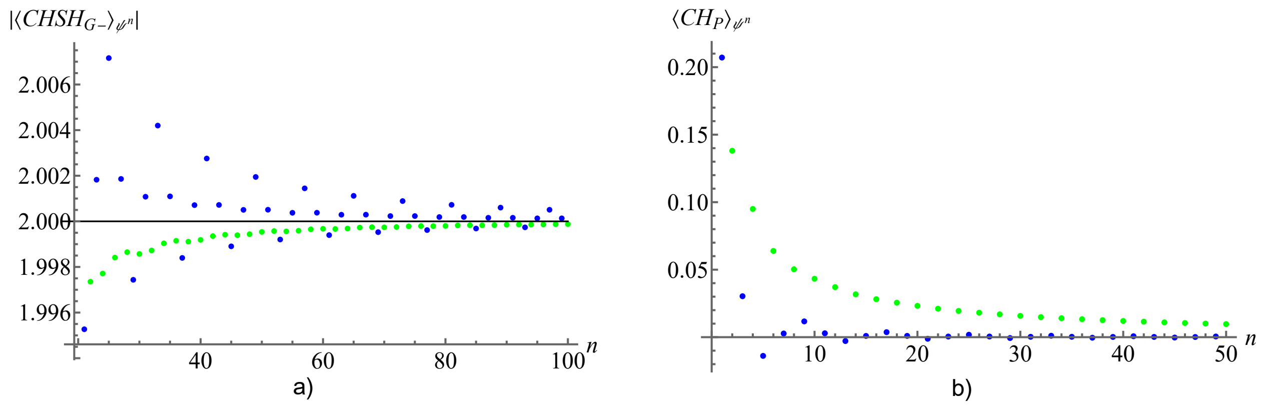

Figure 2. a) $\langle CHSH_{G-}\rangle_{\psi^n}$ versus n. Blue darker dots depict odd n and green dots stand for even n. We can observe two patterns occurring. The first pattern for odd n' oscillates around the bound with decreasing amplitude and period $T = 8$. The second pattern for even n' converges to 2 from below with growing n. This pattern also has an internal structure which is repeatable with $T = 8$ (increase, decrease, increase, increase). Note that only $\left|\psi_-^n\right\rangle$ with odd n violate $CHSH$ inequality for sign Stokes operators. However, not all $\left|\psi_-^n\right\rangle$ for odd n exhibit non-classical correlations. Still, for every n in the odd pattern for which there is no violation (6) we have 3 different odd n' for which violation occurs. b) Quantum predictions of $\langle CH_P\rangle$ for $\left|\psi_-^n\right\rangle$ versus n. Blue darker dots depict odd n and green dots stand for even n. In this case, there are also two patterns. The oscillating odd pattern with the same properties and the convergent even pattern. However, in this case, the even pattern goes to bound from above, and clearly have a higher impact on violation of (16) by the BSV state. This shows that CH inequality (16) is not equivalent to CHSH inequality (6).

where $|\left\langle \psi_-^n \middle| \psi_- \right\rangle|^2 = (n+1)\frac{\tanh^{2n}\Gamma}{\cosh^4\Gamma}$. Fig. 1 shows values of $\langle CHSH_{G-}\rangle_N^{(\Gamma)} - 2$ for $\Gamma = 1,2,3$ and $\Gamma \to \infty$.

We observe that $\langle CHSH_{G-}\rangle_N^{(\Gamma)}$ is a decreasing function of Γ. All calculated values of $\langle CHSH_{G-}\rangle_N^{(\Gamma)}$ exceed 2 and violate the inequality (6). Also $\langle CHSH_{G-}\rangle_N^{(\Gamma)}$ for any given Γ converges to 2 with growing N. Moreover the curve corresponding to $\Gamma \to \infty$ is the most relevant for our analysis because it bounds the $\langle CHSH_{G-}\rangle_N^{(\Gamma)}$ from bellow.

We recall that the quantum prediction for the expectation value of the CHSH inequality (6) consists of two terms: the vacuum term and the non-vacuum term:

$$|\langle \psi_- | CHSH_{G-} | \psi_- \rangle| = |\langle \Omega | CHSH_{G-} | \Omega \rangle| + |\langle CHSH_{G-}\rangle_{nv}| = \frac{2}{\cosh^4\Gamma} + |\langle CHSH_{G-}\rangle_{nv}|. \tag{A2}$$

The non-vacuum term $\langle CHSH_{G-}\rangle_{nv}$ can be written as the weighted average of $\langle CHSH_{G-}\rangle_N^{(\Gamma)}$:

$$|\langle CHSH_{G-}\rangle_{nv}| = \sum_{N=1}^{\infty} \langle CHSH_{G-}\rangle_N^{(\Gamma)} \sum_{n=1+T(N-1)}^{T+T(N-1)} |\left\langle \psi_-^n \middle| \psi_- \right\rangle|^2. \tag{A3}$$

Assuming that there is no change in the pattern of $\langle CHSH\rangle_{\psi^n}$ as n increases (see the discussion below) we can bound from below the value of $|\langle CHSH_{G-}\rangle_{nv}|$ by replacing $\langle CHSH_{G-}\rangle_N^{(\Gamma)}$ with 2:

$$|\langle CHSH_{G-}\rangle_{nv}| \geq \sum_{n=1}^{\infty} 2(n+1)\frac{\tanh^{2n}\Gamma}{\cosh^4\Gamma} = 2(\tanh^2\Gamma + \mathrm{sech}^2\Gamma \tanh^2\Gamma). \tag{A4}$$

Equality due to our assumptions should be only reached in the limit of $\Gamma \to \infty$. This is because in the regime of high values of Γ only terms with high n are significant and $\langle CHSH_{G-}\rangle_N^{(\Gamma)}$ from the assumption reach 2 only when $N \to \infty$. Expression (A4) as expected has an asymptotic value 2. If we add the vacuum term to the RHS of (A4) we obtain constant function equal to 2. Thus, the above reasoning supports the conjecture that for any Γ:

$$|\langle \psi_- | CHSH_{G-} | \psi_- \rangle| \geq 2. \tag{A5}$$

Finally, let us argue why for higher n's the same pattern is expected. Note that $\left|\psi_-^n\right\rangle$ can be written in the following form:

$$\left|\psi_-^n\right\rangle = \frac{1}{n!\sqrt{n+1}}(\hat{a}_H^\dagger \hat{b}_V^\dagger - \hat{a}_V^\dagger \hat{b}_H^\dagger)^n \left|\Omega\right\rangle. \tag{A6}$$

Given some point in the pattern $\langle CHSH_{G-}\rangle_{\psi^n}$ to obtain the next corresponding point in the pattern we only have to apply operator $(\hat{a}_H^\dagger \hat{b}_V^\dagger - \hat{a}_V^\dagger \hat{b}_H^\dagger)^T$ to the state $\left|\psi_-^n\right\rangle$ and normalize it by the factor $\frac{n!\sqrt{n+1}}{(n+T)!\sqrt{n+T+1}}$. To obtain the next k-th corresponding point we have to apply this operator k times. Thus, applying such operator has to preserve some internal symmetries. There is no reason for existence of k such that it suddenly stops to preserve those symmetries. Therefore, the pattern should be continued for any period N.
